# *HDAC7* is overexpressed in human diabetic islets and impairs insulin secretion in rat islets and clonal beta cells

**DOI:** 10.1007/s00125-016-4113-2

**Published:** 2016-10-29

**Authors:** Mahboubeh Daneshpajooh, Karl Bacos, Madhusudhan Bysani, Annika Bagge, Emilia Ottosson Laakso, Petter Vikman, Lena Eliasson, Hindrik Mulder, Charlotte Ling

**Affiliations:** 1 Epigenetics and Diabetes Unit, Department of Clinical Sciences, Lund University Diabetes Centre, Lund University, CRC, 20502 Malmö, Sweden; 2 Molecular Metabolism Unit, Department of Clinical Sciences, Lund University Diabetes Centre, Malmö, Sweden; 3 Diabetes and Endocrinology Unit, Department of Clinical Sciences, Lund University Diabetes Centre, Malmö, Sweden; 4 Islet Cell Exocytosis Unit, Department of Clinical Sciences, Lund University Diabetes Centre, Scania University Hospital, Malmö, Sweden

**Keywords:** Apoptosis, Beta cells, Epigenetic modification, HDAC7, Human pancreatic islets, Insulin secretion, MC1568, Trichostatin A, Type 2 diabetes

## Abstract

**Aims/hypothesis:**

Pancreatic beta cell dysfunction is a prerequisite for the development of type 2 diabetes. Histone deacetylases (HDACs) may affect pancreatic endocrine function and glucose homeostasis through alterations in gene regulation. Our aim was to investigate the role of HDAC7 in human and rat pancreatic islets and clonal INS-1 beta cells (INS-1 832/13).

**Methods:**

To explore the role of HDAC7 in pancreatic islets and clonal beta cells, we used RNA sequencing, mitochondrial functional analyses, microarray techniques, and HDAC inhibitors MC1568 and trichostatin A.

**Results:**

Using RNA sequencing, we found increased *HDAC7* expression in human pancreatic islets from type 2 diabetic compared with non-diabetic donors. *HDAC7* expression correlated negatively with insulin secretion in human islets. To mimic the situation in type 2 diabetic islets, we overexpressed *Hdac7* in rat islets and clonal beta cells. In both, *Hdac7* overexpression resulted in impaired glucose-stimulated insulin secretion. Furthermore, it reduced insulin content, mitochondrial respiration and cellular ATP levels in clonal beta cells. Overexpression of *Hdac7* also led to changes in the genome-wide gene expression pattern, including increased expression of *Tcf7l2* and decreased expression of gene sets regulating DNA replication and repair as well as nucleotide metabolism. In accordance, *Hdac7* overexpression reduced the number of beta cells owing to enhanced apoptosis. Finally, we found that inhibiting HDAC7 activity with pharmacological inhibitors or small interfering RNA-mediated knockdown restored glucose-stimulated insulin secretion in beta cells that were overexpressing *Hdac7*.

**Conclusions/interpretation:**

Taken together, these results indicate that increased HDAC7 levels caused beta cell dysfunction and may thereby contribute to defects seen in type 2 diabetic islets. Our study supports HDAC7 inhibitors as a therapeutic option for the treatment of type 2 diabetes.

**Electronic supplementary material:**

The online version of this article (doi:10.1007/s00125-016-4113-2) contains peer-reviewed but unedited supplementary material, which is available to authorised users.

## Introduction

Data from genome-wide association studies point towards pancreatic beta cell dysfunction as a key defect causing type 2 diabetes [1]. However, the genetic variants identified so far only explain a modest proportion of the estimated heritability of type 2 diabetes, implying that additional factors remain to be discovered [2]. These may include epigenetic mechanisms. Indeed, we and others have identified epigenetic modifications in pancreatic islets, adipose tissue, skeletal muscle and liver from individuals with type 2 diabetes that might be important in the disease pathogenesis [3–8]. Numerous enzymes, including histone deacetylases (HDACs), regulate epigenetic modifications and may thereby affect gene expression and cellular function. A growing body of evidence suggests that HDACs control mammalian pancreatic endocrine cell function and glucose homeostasis [9–11]. For example, mice lacking *Hdac5* exhibit increased beta cell mass [9]. We recently reported decreased DNA methylation and increased gene expression of *HDAC7* in pancreatic islets from human donors with type 2 diabetes [3]. However, the role of HDAC7 in beta cells has not been explored. In the present study, we investigated the functional consequences of *Hdac7* overexpression in beta cells and islets in an effort to dissect its potential role in diabetic islets.

## Methods

### RNA sequencing

Pancreatic islets from 85 non-diabetic and 16 type 2 diabetic donors were obtained from the Human Tissue Lab at EXODIAB/Lund University Diabetes Centre through the Nordic Network for Clinical Islet Transplantation. The selection criteria for non-diabetic donors were no diagnosis of type 2 diabetes and an HbA_1c_ level below 6.0% (52 mmol/mol), as determined by the Mono-S method. The clinical characteristics of the islet donors are shown in Table 1. Parts of this islet cohort have been described previously [12]. High-quality RNA extracted from human islets was used for sequencing with the TruSeq RNA sample preparation kit (Illumina, San Diego, CA, USA) as previously described [12]. This study was approved by the local ethics committee. Informed consent was obtained from pancreatic donors or their relatives.

**Table 1:** Characteristics of human pancreatic islet donors

Variable	No diabetes (*n*=85)	Type 2 diabetes (*n*=16)	*p* value
Male/female (*n*)	52/33	10/6	
HbA_1c_ (%)	5.5±0.4	6.9±1.0	<0.0001
HbA_1c_ (mmol/mol)	46.5±3.6	60.3±10.4	<0.0001
Age (years)	56.1±11.1	59.9±11.6	0.24
BMI (kg/m^2^)	25.6±3.1	28.2±4.3	0.03
GSIS (ng islet^–1^ h^–1^) at 16.7 mmol/l glucose	0.92±0.95	0.42±0.36	0.05

Data are presented as means ± SD, unless otherwise indicated

The Mann–Whitney *U* test was used for statistical analysis

### Rat islet isolation and culture

Pancreatic islets from 8- to 10-week-old male Wistar rats (Taconic, Lille Skensved, Denmark) were isolated by collagenase digestion and hand-picked under a stereo microscope [13]. The isolated islets were precultured for 24 h before adenoviral transduction in RPMI 1640 with UltraGlutamine (Lonza, Vallensbaek, Denmark) supplemented with 10% newborn calf serum (Biological Industries, Kibbutz Beit Haemek, Israel), 100 U/ml penicillin and 100 μg/ml streptomycin (Life Technologies, Paisley, UK) in 5% CO_2_ at 37°C. All animal experiments were approved by the local ethics committee and performed in accordance with the Guide for the Care and Use of Laboratory Animals [14].

### Overexpression of *Hdac7* in rat islets and clonal beta cells

An adenoviral vector for *Hdac7* overexpression, Ad-GFP-CMV-ratHdac7, and a control vector conferring only green fluorescent protein expression, Ad-GFP-CMV, were made by Vector Biolabs (Philadelphia, PA, USA). Isolated rat islets were infected with 50,000 virus particles/islet. The rat clonal beta cell line INS-1 832/13 was transfected with a pcDNA3.1 expression vector containing the cDNA sequence of rat *Hdac7* (Genscript, Piscataway, NJ, USA) or the empty vector (control) by using Lipofectamine LTX (Life Technologies). Experiments were performed 48 h after transduction/transfection, unless stated otherwise.

### PCR and western blot

mRNA expression of *Hdac7* and *Tcf7l2* was analysed using TaqMan assays and related to expression of *Ppia* (Life Technologies) by quantitative real-time (q)PCR and the ΔΔC_t_ method. To verify overexpression of HDAC7 protein, clonal beta cells were transfected with haemagglutinin-tagged cDNA for *Hdac7* and lysed in RIPA buffer (50 mmol/l Tris, pH 7.6, 150 mmol/l NaCl, 0.1% SDS, 0.5% sodium deoxycholate, 1% Triton-X100, protease inhibitor cocktail; Sigma-Aldrich, St Louis, MO, USA), and boiled with sample buffer (60 mmol/l Tris, pH 6.8, 10% glycerol, 2% SDS, 10% β-mercaptoethanol, bromophenol blue). Samples were separated on Mini-PROTEAN TGX gels (Bio-Rad, Hercules, CA, USA) and transferred onto Hybond-LFP PVDF membranes (GE Healthcare, Piscataway, NJ, USA). Protein expression was detected using a rabbit haemagglutinin tag (Abcam, Cambridge, UK; diluted 1:4000) and mouse β-actin (Sigma-Aldrich; diluted 1:10,000) antibodies, and secondary DyLight 680/800 conjugated goat antibodies (Thermo Scientific, Rockford, IL, USA; diluted 1:15,000), all validated by the respective suppliers. Blots were scanned using an Odyssey imaging system (LI-COR, Lincoln, NE, USA).

### Insulin secretion and content

Glucose-stimulated insulin secretion (GSIS) was examined in isolated rat islets. For each condition (*Hdac7* overexpression and control), 24 islets (three islets per well) were transferred to Krebs–Ringer HEPES buffer (115 mmol/l NaCl, 4.7 mmol/l KCl, 2.6 mmol/l CaCl_2_, 1.2 mmol/l KH_2_PO_4_, 1.2 mmol/l MgSO_4_, 10 mmol/l HEPES, 0.2% BSA, 2 mmol/l glutamine, 5 mmol/l NaHCO_3_, 100 U/ml penicillin, 100 μg/ml streptomycin, pH 7.4) containing 2.8 mmol/l glucose and incubated 30 min prior to the GSIS experiment. Islets were then exposed to basal (2.8 mmol/l) or stimulatory (16.7 mmol/l) glucose for 60 min. The buffer was then collected and insulin secretion was determined using ELISA (Mercodia, Uppsala, Sweden) and normalised to insulin content.

Insulin secretion was also measured in transfected clonal beta cells at 2.8 and 16.7 mmol/l glucose levels in a secretion assay buffer (SAB) with normal (5.9 mmol/l) as well as elevated (35 mmol/l) K^+^ concentrations, as previously described [15]. In addition, two HDAC inhibitors, MC1568 (1 μmol/l; Sigma-Aldrich) [16] and trichostatin A (TSA) (0.625 μmol/l; Sigma-Aldrich) [17] were added to the culture medium 24 h before secretion experiments, as indicated. Insulin was measured in the supernatant fraction using an insulin RIA (Siemens Diagnostics, Erlangen, Germany) or insulin ELISA and normalised to total protein, as determined by a bicinchoninic acid assay (Thermo Scientific). Total insulin content was determined after acid ethanol extraction and normalised to protein content.

### Mitochondrial respiration

The Extracellular Flux Analyzer XF24 (Seahorse Bioscience, North Billerica, MA, USA) was used to monitor the oxygen consumption rate (OCR) as described elsewhere [18]. Clonal beta cells were cultured and transfected as above on XF24 microplates coated with poly-d-lysine (10 μg/ml) at 200,000 cells/well prior to analysis. Data were normalised to total protein.

### ATP measurement

Clonal beta cells were starved for 2 h in SAB containing 2.8 mmol/l glucose. Next, the cells were treated with SAB containing either 2.8 or 16.7 mmol/l glucose for 15 min, washed in PBS, lysed in water and kept on dry ice for 15 min. Lysates were thawed, sonicated for 15 s and used for ATP and total protein measurements. The level of ATP was monitored using an ATP Kit SL (BioThema, Handen, Sweden) according to the manufacturer’s instructions.

### Microarray mRNA expression analysis

RNA was extracted from transfected clonal beta cells and the quantity and quality were assessed using the NanoDrop spectrophotometer ND-1000 (Thermo Fisher Scientific, Wilmington, DE, USA) and 2100 Bioanalyzer (Agilent, Santa Clara, CA, USA), respectively. Whole-transcript microarray analysis was conducted on the WT Gene 2.0 ST Array (Affymetrix, Santa Clara, CA, USA) by the Swegene Center for Integrative Biology at Lund University. mRNA expression data were obtained from a total of 30,619 probe sets, representing 20,173 annotated transcripts and 19,603 unique genes. The Robust Multi-array Average method was used for background correction, data normalisation and probe summarisation.

### Gene Set Enrichment Analysis

Gene Set Enrichment Analysis (GSEA) [19, 20] (www.broad.mit.edu/GSEA) version 4.0 was used to identify enriched gene sets of the microarray data using the Kyoto Encyclopedia of Genes and Genomes (KEGG). Probe sets pertaining to transcripts were ranked based on *t* statistics values from paired *t* tests.

### Cell number, apoptosis and proliferation assays

Clonal beta cells were seeded and transfected on 96-well plates. Cell number was determined by crystal violet staining, as previously described [3]. Caspase-3 and -7 activity as a measure of apoptosis were assessed using Apo-ONE Homogeneous Caspase-3/7 Assay (Promega, Madison, WI, USA) according to the manufacturer’s protocol. Cell proliferation was measured with the Cell Proliferation ELISA, BrdU kit (Roche Applied Sciences, Mannheim, Germany). BrdU labelling solution was added to the cells 24 h post-transfection and cells were cultured for another 24 h prior to absorbance measurement with a Tecan Infinite M200 Pro plate reader (Tecan Group, Männedorf, Switzerland).

### Silencing of *Hdac7* and *Tcf7l2* in INS-1 832/13 cells

INS-1 832/13 beta cells were transfected with *Hdac7* plasmid as described above and cotransfected with small interfering (si)RNAs targeting *Hdac7* (*siHdac7*, 12.5 nmol/l) or *Tcf7l2* (*siTcf7l2*, 25 nmol/l) (ON-TARGETplus siRNA–SMARTpool; Dharmacon, Heidelberg, Germany) using the DharmaFECT I siRNA Transfection Reagent (Thermo Scientific). A non-targeting siRNA (5ʹ-GAGACCCUAUCCGUGAUUAUU-3ʹ) was used as negative control. Silencing was validated by qPCR, as described above.

### Chromatin immunoprecipitation (ChIP)

ChIP followed by ChIP-qPCR was performed on *Hdac7*- and control-transfected INS-1 832/13 beta cells to test for enrichment of histone 3 lysine 27 acetylation (H3K27ac) at the *Tcf7l2*, *Gapdh* and *Ldha* genes (see electronic supplementary material [ESM] Methods and ESM Table 1).

### Statistics

RNA sequencing data were analysed using a Mann–Whitney *U* test. Wilcoxon signed-rank tests were used to analyse the rat islet and clonal beta cell data. False discovery rate analysis was used to correct for multiple testing.

## Results

### *HDAC7* expression is increased in pancreatic islets from human donors with type 2 diabetes

**Fig. 1:**
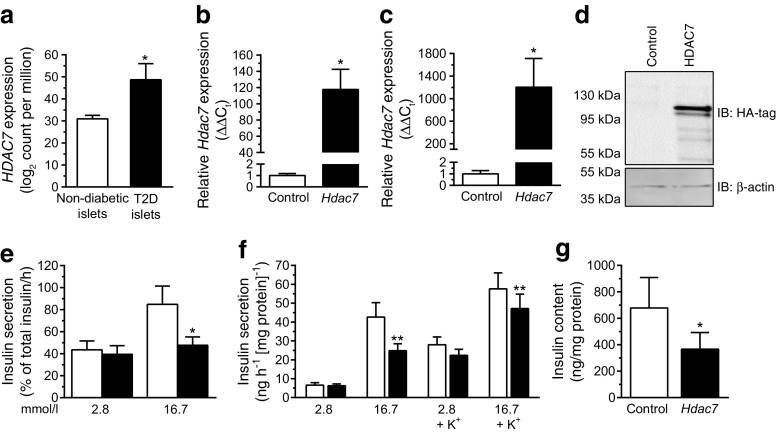
*Hdac7* overexpression impaired insulin secretion and content. (**a**) *HDAC7* expression, as measured by RNA sequencing, was higher in human pancreatic islets from donors with type 2 diabetes (*n* = 16) compared with non-diabetic controls (*n* = 85). (**b**) Transduction of isolated rat islets with an adenoviral vector encoding *Hdac7* led to significant overexpression of *Hdac7* mRNA. (**c**, **d**) Transfection of clonal beta cells with a pcDNA3.1 expression plasmid containing *Hdac7* resulted in elevated mRNA (**c**) and protein (**d**) levels (*n* = 6 and *n* = 3, respectively). (**e**) *Hdac7* overexpression in rat islets resulted in reduced GSIS (*n* = 6). White bars, control; black bars, *Hdac7*. (**f**) Insulin secretion in response to 2.8 and 16.7 mmol/l glucose with or without depolarising concentrations of K^+^ in clonal beta cells overexpressing *Hdac7* compared with control transfected cells (*n* = 9). White bars, control; black bars, *Hdac7*. (**g**) *Hdac7* overexpression resulted in reduced insulin content in clonal beta cells (*n* = 6). Data are presented as means ± SEM. *p < 0.05, **p < 0.01. RNA sequencing data were analysed with a Mann–Whitney *U* test, and Wilcoxon signed-rank tests were used to analyse the rat islet and clonal beta cell data. HA-tag, haemagglutinin-tag; IB, immunoblot antibody; T2D, type 2 diabetes

Using RNA sequencing, we found increased *HDAC7* expression in pancreatic islets from donors with type 2 diabetes compared with non-diabetic controls (Fig. 1a), confirming our previous finding [3]. There were also significant differences in GSIS at 16.7 mmol/l glucose (*p* = 0.05), HbA_1c_ (*p* < 0.0001) and BMI (*p* = 0.03) between the non-diabetic and diabetic donors (Table 1). However, there was no significant difference in age (*p* = 0.24) between the two groups. In addition, the expression level of *HDAC7* correlated negatively with GSIS in human islets (*r* = –0.41, *p* < 0.004).

### Impaired GSIS in rat islets and clonal beta cells overexpressing *Hdac7*

The expression data on *HDAC7* from human islets do not resolve whether changes are a primary or secondary phenomenon. To address this question, and mimic the situation in human type 2 diabetic islets, we overexpressed *Hdac7* in isolated rat islets and in INS-1 832/13 beta cells (Fig. 1b–d). Increased HDAC7 expression resulted in reduced GSIS at 16.7 mmol/l glucose in both rat islets and clonal beta cells (Fig. 1e,f). In addition, *Hdac7* overexpression had nominal effects on insulin secretion in response to only the membrane-depolarising agent KCl (*p* = 0.054 at 2.8 mmol/l glucose), but there was no effect on the basal secretion at 2.8 mmol/l glucose (Fig. 1f). Furthermore, the insulin content was reduced in *Hdac7*-overexpressing beta cells (Fig. 1g), but unaffected in rat islets (data not shown).

### *Hdac7* overexpression impairs mitochondrial function

**Fig. 2:**
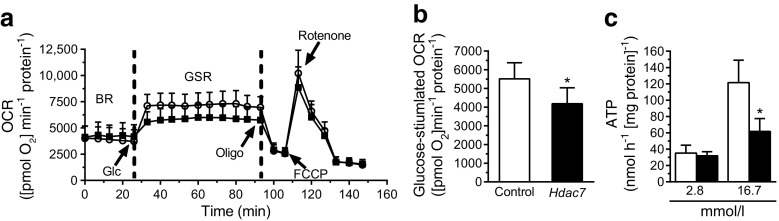
*Hdac7* overexpression resulted in mitochondrial dysfunction. (**a**) The OCR in clonal beta cells overexpressing *Hdac7* and control cells (*n* = 5). The OCR was measured in the presence of 2.8 mmol/l glucose (basal respiration, BR) and then after the sequential addition of 16.7 mmol/l glucose (Glc; glucose-stimulated respiration, GSR), 4 μg/ml oligomycin (oligo), 4 μmol/l carbonyl cyanide *p*-trifluoromethoxyphenylhydrazone (FCCP) and 1 μmol/l rotenone. White circles, control; black squares, *Hdac7*. (**b**) The glucose-stimulated OCR was significantly decreased in *Hdac7*-overexpressing beta cells compared with control cells (*n* = 5). *p < 0.001 as analysed by a paired *t* test. (**c**) Cellular ATP levels were reduced in *Hdac7*-overexpressing clonal beta cells (*n* = 6). White bars, control; black bars, *Hdac7*. Data are presented as means ± SEM. *p < 0.05, as analysed by Wilcoxon signed-rank tests

A possible explanation for the reduced GSIS in *Hdac7*-overexpressing beta cells is deficient mitochondrial function. Indeed, we found mitochondrial respiration at elevated glucose levels to be reduced in *Hdac7*-overexpressing cells (Fig. 2a, b). This was reflected by reduced glucose-stimulated ATP levels in these cells (Fig. 2c), as well as by reduced oligomycin-sensitive respiration (i.e. ATP turnover; Fig. 2a).

### Altered gene expression and cell number in beta cells overexpressing *Hdac7*

As HDAC7 may alter gene expression, we investigated the genome-wide expression pattern in *Hdac7*-overexpressing and control clonal INS-1 832/13 beta cells. We found 1171 differentially expressed genes at *p* < 0.05 (548 genes upregulated and 623 genes downregulated), but no individual gene, except *Hdac7*, had a false discovery rate of less than 5% (ESM Table 2). However, based on the impaired mitochondrial function in *Hdac7*-overexpressing beta cells, we specifically examined whether the expression of genes with known roles in mitochondrial metabolism was decreased (*p* < 0.05) in these cells. Indeed, *Sdhc*, *Ndufa7* and *Atp5g3*, which are involved in the tricarboxylic acid cycle and the electron transport chain, exhibited decreased expression (ESM Table 2). Furthermore, the expression of *Pcsk1*, which is involved in the processing of proinsulin to insulin, was slightly reduced in *Hdac7*-overexpressing cells.

To identify biological pathways with altered expression in *Hdac7*-overexpressing beta cells, we next used GSEA. This analysis yielded eight significant gene sets with downregulated expression in beta cells overexpressing *Hdac7* (*q* < 0.05, Table 2). These included pathways involved in DNA replication and repair, transcription and nucleotide metabolism, as well as protein folding, sorting and degradation. However, we found no significant gene sets with upregulated expression.

**Table 2:** Genes contributing to the enrichment scores of GSEA for gene sets downregulated in *Hdac7*-overexpressing vs control beta cells

Gene set ID and name	Genes related to the enrichment score	Regulated genes/total	Enrichment score	*p* value	*q* value
RN03030 DNA replication	*Dna2*, *Lig1*, *Mcm2*, *Mcm6*, *Mcm7*, *Pcna*, *Pola2*, *Pold2*, *Pole*, *Pole2*, *Prim2*, *Rfc2*, *Rfc3*, *Rfc5*, *Rnaseh1*, *Rnaseh2a*, *Rpa1*, *Rpa2*, *Rpa3*, *Ssbp1*	20/31	0.6334	<0.001	<0.001
RN03020 RNA polymerase	*Polr1a*, *Polr1b*, *Polr1c*, *Polr1e*, *Polr2a*, *Polr2b*, *Polr2c*, *Polr2e*, *Polr2f*, *Polr2i*, *Polr3b*, *Polr3d*, *Polr3h*, *Polr3k*	14/23	0.6838	<0.001	<0.001
RN03040 Spliceosome	*Acin1*, *Alyref*, *Bcas2*, *Ccdc12*, *Cherp*, *Ddx23*, *Ddx39b*, *Ddx46*, *Dhx15*, *Dhx38*, *Eftud2*, *Hnrnpu*, *Lsm3*, *Lsm4*, *Lsm5*, *Lsm6*, *Magoh*, *Magohb*, *Ncbp1*, *Phf5a*, *Ppie*, *Ppih*, *Ppil1*, *Pqbp1*, *Prpf3*, *Prpf4*, *Prpf40a*, *Prpf8*, *Puf60*, *Rbm8a*, *Sf3a1*, *Sf3a2*, *Sf3a3*, *Sf3b3*, *Sf3b4*, *Sf3b5*, *Slu7*, *Snrnp27*, *Snrnp40*, *Snrnp70*, *Snrpb*, *Snrpd1*, *Snrpf*, *Srsf1*, *Srsf4*, *Srsf5*, *Srsf7*, *Srsf9*, *Thoc2*, *Thoc3*, *Usp39*, *Wbp11*, *Zmat2*	54/98	0.5070	<0.001	<0.001
RN03430 Mismatch repair	*Exo1*, *Lig1*, *Mlh1*, *Mlh3*, *Msh2*, *Pcna*, *Pold2*, *Rfc2*, *Rfc3*, *Rfc5*, *Rpa1*, *Rpa2*, *Rpa3*, *Ssbp1*	14/21	0.6394	<0.001	0.0024
RN03050 Proteasome	*Psma1*, *Psma2*, *Psma5*, *Psma6*, *Psmb1*, *Psmb2*, *Psmb3*, *Psmb7*, *Psmc2*, *Psmc3*, *Psmc5*, *Psmc6*, *Psmd1*, *Psmd2*, *Psmd3*, *Psmd11*, *Psmd12*, *Psmd13*, *Psmd14*, *Shfm1*	20/40	0.5284	0.0018	0.0035
RN03420 Nucleotide excision repair	*Ddb1*, *Ercc1*, *Gtf2h3*, *Gtf2h5*, *Pcna*, *Pole*, *Pole2*, *Rbx1*, *Rfc2*, *Rfc5*, *Rpa1*, *Rpa2*, *Rpa3*	13/40	0.5111	<0.001	0.0096
RN03440 Homologous recombination	*Eme1*, *Mre11a*, *Mus81*, *Pold2*, *Rad51*, *Rad51c*, *Rad54b*, *Rpa1*, *Rpa2*, *Rpa3*, *Shfm1*, *Ssbp1*	12/23	0.5638	0.0018	0.0164
RN00240 Pyrimidine metabolism	*Cad*, *Ctps1*, *Dck*, *Dtymk*, *Dut*, *Entpd5*, *Itpa*, *Nme1*, *Nme3*, *Nt5c1a*, *Nt5c3a*, *Nt5m*, *Pola2*, *Pold2*, *Pole*, *Pole2*, *Polr1a*, *Polr1b*, *Polr1c*, *Polr1e*, *Polr2a*, *Polr2b*, *Polr2c*, *Polr2e*, *Polr2f*, *Polr2i*, *Polr3b*, *Polr3d*, *Polr3h*, *Polr3k*, *Prim2*, *Rrm2*, *Txnrd1*, *Tyms*, *Umps*, *Upb1*	36/79	0.4141	<0.001	0.0290

**Fig. 3:**
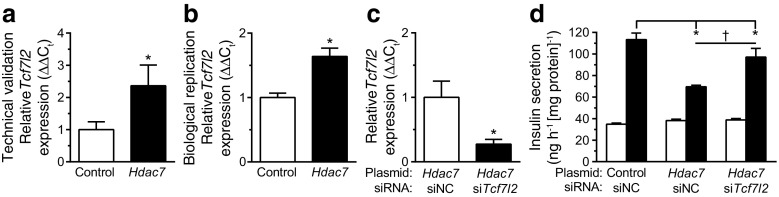
*Hdac7* overexpression impaired insulin secretion partly through increased *Tcf7l2* expression. qPCR was used to technically (**a**, *n* = 6) and biologically (**b**, *n* = 4) validate the increased expression of *Tcf7l2* in *Hdac7*-overexpressing clonal beta cells. *p < 0.05. Silencing *Tcf7l2* (**c**) partially restored GSIS in *Hdac7*-overexpressing beta cells (**d**). White bars, 2.8 mmol/l glucose; black bars, 16.7 mmol/l glucose; *n* = 6. *p < 0.05 vs siRNA negative control (siNC); ^†^ *p* < 0.05 vs *Hdac7* siNC. Data are presented as means ± SEM

In addition, *Tcf7l2* was one of the most significant genes in the microarray analysis and was upregulated in beta cells overexpressing *Hdac7* (ESM Table 2). Interestingly, *TCF7L2* single-nucleotide polymorphisms have shown the strongest association with type 2 diabetes in genome-wide association studies, and *TCF7L2* has been proposed to regulate islet function [21]. We used qPCR to technically validate the upregulation of *Tcf7l2* in RNA isolated from the *Hdac7*-overexpressing beta cells used for the microarray analysis (Fig. 3a). We could also biologically replicate these data in a different set of transfected cells. (Fig. 3b). We next tested whether restoring the expression of *Tcf7l2* in *Hdac7-*overexpressing cells, by cotransfecting these cells with an siRNA against *Tcf7l2*, had any impact on insulin secretion. Interestingly, knockdown of *Tcf7l2* partially reversed the insulin secretion impairment in *Hdac7*-overexpressing cells (Fig. 3c, d).

Based on the altered expression of gene sets regulating DNA replication and nucleotide metabolism, we next tested whether beta cell proliferation and/or apoptosis were altered when *Hdac7* was overexpressed. Increased HDAC7 levels slightly, but significantly, reduced the number of beta cells (ESM Fig. 1a). Moreover, caspase-3 and -7 activity was increased in *Hdac7*-overexpressing cells, reflecting increased apoptosis, whereas BrdU-incorporation was unchanged, suggesting that cell proliferation was not affected (ESM Fig. 1b,c). In isolated rat islets, however, short-term overexpression of *Hdac7* did not result in enhanced apoptosis (ESM Fig. 1d).

### Histone acetylation

To investigate whether histone acetylation was affected by *Hdac7* overexpression, we performed ChIP for the H3K27ac mark, which is associated with promoters and enhancer regions of active genes. We performed ChIP-qPCR for *Tcf7l2*, as well as for *Gapdh* and *Ldha* as positive and negative controls, respectively, since *Gapdh* is highly expressed and *Ldha* is expressed at very low levels, if at all, in beta cells. As expected, the *Gapdh* promoter was highly enriched for H3K27ac, whereas the mark was virtually absent in *Ldha*. There was no significant difference in H3K27ac enrichment between the *Hdac7*-overexpressing and control cells for *Gapdh* or *Ldha* (ESM Fig. 2). Furthermore, *Tcf7l2* also failed to show significant differences in H3K27ac enrichment between control and *Hdac7*-overexpressing cells (ESM Fig. 2), suggesting other regulatory mechanisms for the differential *Tcf7l2* expression.

### HDAC7 inhibition restores GSIS in beta cells overexpressing *Hdac7*

**Fig. 4:**
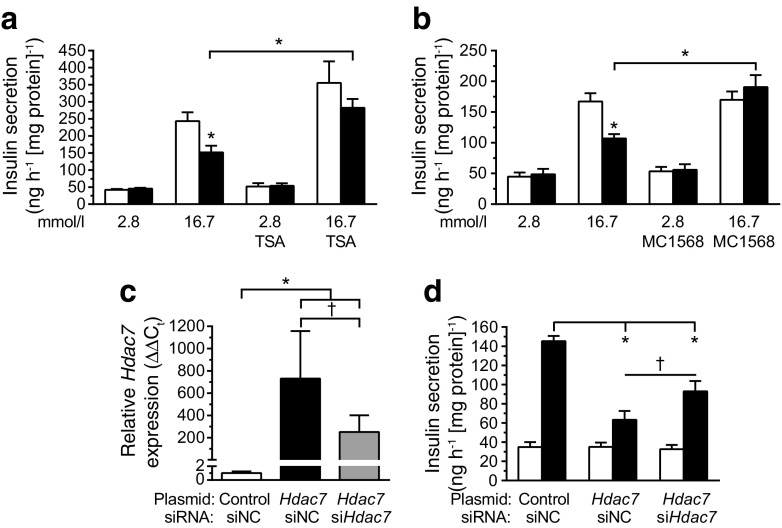
Inhibition of HDAC7 rescued impaired insulin secretion. (**a**) TSA (0.625 μmol/l) restored GSIS in *Hdac7*-overexpressing clonal beta cells, while the effect on control cells did not reach significance (*p* = 0.0625) (*n* = 6). White bars, control; black bars, *Hdac7*. (**b**) MC1568 (1 μmol/l) restored GSIS in *Hdac7*-overexpressing clonal beta cells (*n* = 6). White bars, control; black bars, *Hdac7*. (**c**) *Hdac7* silencing (60%) in *Hdac7-*overexpressing clonal beta cells was confirmed by qPCR (*n* = 6). (**d**) Silencing *Hdac7* in cotransfected *Hdac7-*overexpressing beta cells partially restored GSIS (*n* = 6). White bars, 2.8 mmol/l glucose; black bars, 16.7 mmol/l glucose. Data are presented as means ± SEM. *p < 0.05; ^†^ *p* < 0.05 vs *Hdac7* siRNA negative control (siNC)

In an attempt to rescue the *Hdac7*-associated beta cell dysfunction, and to test if HDAC7 may be a potential target for novel type 2 diabetes therapies, we treated *Hdac7-*overexpressing beta cells with two different HDAC inhibitors: TSA (an inhibitor of class I and class II HDACs) and MC1568 (an inhibitor of class II HDACs) [16, 17]. TSA treatment nominally increased GSIS in the beta cells transfected with the control vector (*p* = 0.0625). However, the effect was greater on *Hdac7*-overexpressing beta cells and the negative impact of HDAC7 on GSIS was no longer apparent (Fig. 4a). MC1568 treatment also completely reversed the negative effect of *Hdac7* overexpression on GSIS, but without affecting insulin secretion in the control cells (Fig. 4b).

Finally, as a more specific approach, we tested whether cotransfecting the *Hdac7*-overexpressing cells with an siRNA targeting *Hdac7* could rescue the secretory defect seen in Fig. 1f. The siRNA resulted in 60% lower *Hdac7* expression compared with cells transfected with the *Hdac7* overexpression plasmid alone (Fig. 4c). This resulted in a partial rescue of the decreased GSIS in *Hdac7*-overexpressing beta cells (Fig. 4d). The fact that the rescue was not complete is very likely explained by *Hdac7* still being significantly overexpressed compared with control transfected cells (Fig. 4c).

## Discussion

In this study, we have shown that *HDAC7* is upregulated in type 2 diabetic human islets, and impairs insulin secretion and mitochondrial function and induces apoptosis when overexpressed in clonal beta cells. Importantly, insulin secretion was restored when *Hdac7*-overexpressing beta cells were treated with HDAC inhibitors, supporting the use of such compounds in the treatment of diabetes.

HDAC inhibitors are currently used for the treatment of epilepsy and cancer [22, 23], and HDACs might also be interesting pharmacological targets for type 2 diabetes. Indeed, work from Mandrup-Poulsen and colleagues has shown that several HDACs, including HDAC1–3, HDAC6 and HDAC11, regulate beta cell function [24–26]. Although these studies imply that increased HDAC levels may contribute to diabetes via hyperglycaemia and cytokine-induced toxicity, they did not identify HDAC7 as a target for the disease. Using microarray techniques, we recently found decreased DNA methylation and increased expression of *HDAC7* in pancreatic islets from donors with type 2 diabetes [3], supporting a possible role for this enzyme in beta cell dysfunction and diabetes. Importantly, through the use of RNA sequencing in a larger islet cohort, we also found increased *HDAC7* levels in diabetic islets in the present study. We also found a negative correlation between *HDAC7* expression and insulin secretion from human islets cultured in vitro. Using functional experiments in clonal beta cells, we could further dissect how HDAC7 contributes to impaired insulin secretion. These experiments suggest that HDAC7 impairs mitochondrial function, increases beta cell apoptosis and increases *Tcf7l2* expression. An impaired capacity to increase ATP production in beta cells in response to elevated glucose levels is indeed an important defect seen in diabetes, resulting in reduced insulin secretion [27–29]. The effect of *Hdac7* overexpression on mitochondrial function may be mediated by the altered transcription of genes involved in metabolic processes. Indeed, we found decreased expression of genes involved in the tricarboxylic acid cycle and electron transport chain. It could also be due to interactions with and/or deacetylation of non-histone proteins affecting mitochondrial function. For example, HDAC7 has been shown to interact with and increase the transcriptional activity of hypoxia-inducible factor 1α [30], a known regulator of mitochondrial metabolism [31]. *TCF7L2* is known as the ‘top’ diabetes gene, and the genotype increasing the risk for type 2 diabetes is also associated with increased *TCF7L2* expression in human islets [21]. Interestingly, in the current study we found reduced insulin secretion and content in beta cells with increased *Hdac7* and *Tcf7l2* levels. Our findings are also in line with those of a previous study, showing that inhibition of HDACs enhances mitochondrial function and oxidative metabolism in muscle and adipose tissue [32]. Furthermore, a reduced number of functional beta cells can contribute to type 2 diabetes. Our data showing a decreased beta cell number due to increased apoptosis with *Hdac7* overexpression are also supported by a previous study in cancer cells, which demonstrated that ectopic expression of *HDAC7* promotes apoptosis and inhibits tumour growth [33].

In addition, there is evidence that HDACs may be promising pharmacological targets in multifactorial diseases [34]. To investigate if HDAC7 inhibition restores insulin secretion, we treated *Hdac7*-overexpressing beta cells with two different HDAC inhibitors. Indeed, TSA treatment restored insulin secretion in *Hdac7*-overexpressing beta cells. However, it also nominally increased insulin secretion in control cells, potentially due to effects on enzymes other than HDAC7. We therefore also used MC1568, a selective inhibitor of class II HDACs [16]. Importantly, MC1568 restored insulin secretion in *Hdac7*-overexpressing beta cells, but had no effect on control cells. These experiments support the development of a HDAC7-specific inhibitor for potential use in the treatment of diabetes.

The reduced methylation and increased expression of *HDAC7* seen in type 2 diabetic islets may be due to environmental and/or genetic factors. We have previously published studies on clonal beta cells and human islets treated with elevated levels of palmitate (i.e. a type 2 diabetes-like treatment), but found no differences in *HDAC7* expression in these studies [18, 35]. This does not, however, exclude elevated palmitate levels as a cause of increased *HDAC7* expression, as such changes may need longer than the 48 h treatment we used to be established. Furthermore, we recently performed a methylation quantitative trait loci study in human pancreatic islets to identify genetic variants that influence DNA methylation and expression [36]. However, we did not find any single-nucleotide polymorphisms associated with altered expression or methylation of *HDAC7*. Other environmental factors may, however, alter the methylation and expression of *HDAC7* in islets.

In addition, the decreased methylation and increased expression of *HDAC7* may occur as a result of environmental insults during embryonic development. This may potentially predispose individuals to diabetes by affecting the number of beta and/or alpha cells in the mature pancreas. Interestingly, it is known that the development of endocrine cells is controlled by HDACs, and HDAC inhibition by TSA has been reported to increase the number of neurogenin-3-expressing progenitor cells [37]. In addition, selective inhibition of class IIa HDACs (HDAC4, -5, -7 and -9) has been reported to increase the pool of beta and delta cells [9].

Pancreatic islets contain cell types other than beta cells, and it is possible that the increased expression of *HDAC7* in diabetic islets stems from differential expression of *HDAC7* in the different islet cell types, in combination with altered cellular composition of the islets. Unfortunately, to our knowledge, no data that can conclusively resolve this issue exists, and we have been unsuccessful in our attempts to generate this type of data owing to limitations in islet material. However, publicly available expression data on isolated human alpha and beta cells, the two major islet cell types, show that the expression of *HDAC7* does not differ between the two cell types [38], and our own published data found that islet cell composition did not differ between diabetic and non-diabetic donors in a subset of our cohort [3].

Furthermore, other HDACs may contribute to type 2 diabetes. In fact, the expression of *HDAC1* and *HDAC11* was increased and decreased, respectively, in our previously analysed cohort of islets from diabetic and control donors (T. Dayeh and C. Ling, unpublished data). However, these changes were of smaller relative magnitude compared with the changes in *HDAC7*, and these enzymes have already been somewhat investigated in beta cells [25, 26]. It is also possible that increased HDAC7 levels may alter the expression of other HDACs. In our *Hdac7*-overexpressing beta cells, we could see that the expression of *Hdac5* was slightly reduced (*p* = 0.045). The present study did not investigate whether this has any functional effects on beta cells.

In conclusion, our study identifies HDAC7 as an enzyme that regulates beta cell function and number, and which is upregulated in human diabetic islets. It also supports further development of HDAC7 inhibitors for diabetes therapy.

## Electronic supplementary material

Below is the link to the electronic supplementary material.

ESM (PDF 506 kb)

ESM Table 2 (XLSX 646 kb)

## Abbreviations

ChIP: Chromatin immunoprecipitation
GSEA: Gene Set Enrichment Analysis
GSIS: Glucose-stimulated insulin secretion
H3K27ac: Histone 3 lysine 27 acetylation
HDAC: Histone deacetylase
OCR: Oxygen consumption rate
qPCR: Quantitative real-time PCR
SAB: Secretion assay buffer
siRNA: Small interfering RNA
TSA: Trichostatin A
