# Supplementary material for: HDAC7 is overexpressed in human diabetic islets and impairs insulin secretion in rat islets and clonal beta cells
Source: Diabetologia. 2016 Oct 29;60(1):116–25. doi: 10.1007/s00125-016-4113-2 (PMC6518079; doi:10.1007/s00125-016-4113-2)
Supplement: Supplementary file 1 — (PDF 506 kb) [file 125_2016_4113_MOESM1_ESM.pdf]

ESM METHODS

Chromatin Immunoprecipitation

$6*10^6$ 832/13 INS-1 cells were seeded on 100mm dishes and transfected with control or *Hdac7* plasmids as described in the main text. Chromatin immunoprecipitation (ChIP) was performed 48h post transfection as described in the iDeal ChIP-seq kit (Diagenode, Liege, Belgium). Cells were collected, washed twice with PBS and cross-linked in PBS with 1% formaldehyde for 8min. The reaction was stopped by addition of glycine and 5min incubation at room temperature. The cells were collected by centrifugation at 240g for 10min at 4°C, lysed in lysis buffer and sheared with shearing buffer using Bioruptor (Diagenode, Liege, Belgium). Immunoprecipitation was conducted using anti-H3K27ac (ab4729, Abcam) and IgG (12-370, Millipore) antibodies accompanied with DiaMag Protein A-coated magnetic beads. 1µl of chromatin was saved for determination of input. After incubation overnight, the beads were sequentially washed with wash buffer 1, 2, 3, and 4, and the chromatin was eluted in elution buffer. Reverse crosslinking was performed overnight at 65°C with buffer iE2. The DNA was isolated using magnetic beads and used for qPCR analysis.

ChIP-quantitative PCR

ChIP-qPCR was performed to test the enrichment of histone modification H3K27ac at the *Tcf7l2*, *Gapdh* and *Ldha* genes. While seven different PCR primer pairs targeting sequences within -2 kb to +4kb of the transcription start site were used for *Tcf7l2*, one primer pair located near the respective transcription start site was used for Gapdh and *Ldha*. The primers were designed using Primer3Plus and are presented in Supplementary Table 2. The ChIP-qPCR was performed using Power SYBR green (Life technologies) in 10µl reactions. The percentage of recovery over input was calculated using the formula; % of recovery = 2^ (Ctinput – Ctsample).

ESM Table 1. Primers used for the analysis of ChIP-qPCR

**Primer id**	**Forward Primer (5'-3')**	**Reverse Primer (5'-3')**
Tcf7l2 -2kb	ATCATACCTGCCCACTGCTC	TCTCATGAGGAGGGATGACC
Tcf7l2 -0.8kb	GGGAGAAGGCAGTGGAGAAT	TGCACGTGCTTGTTGTATTG
Tcf7l2 -0.25kb	AAGAGCAGCATCTTGGGTTC	TAAAAGTGGGAGGCAGTTGG
Tcf7l2 TSS	CTCACCGCATATCCCTTTGT	AGAGGTGTGAGTGGGTGGAC
Tcf7l2 0.8kb	CACCCGGGGAGACTTAATTT	GGACAGGACAGGGATGAAAA
Tcf7l2 2.0kb	GTCAATCACCTCAGCCCATC	AGGACTGTCCCAGCCTTCTT
Tcf7l2 4.0kb	TGCGCTTCCTTGTTTATCTG	AAAGAAACCGAAACCACACG
Gapdh -0.8kb	TCGCGGTACACAAGCCAGGAC	GGCCCTTCGCAGCCCCATTT
Ldha -0.25kb	TTTTACGGAGAAGGAAGCGGAGGA	AATGGCCAGCCGGACATGCT

ESM Figure 1

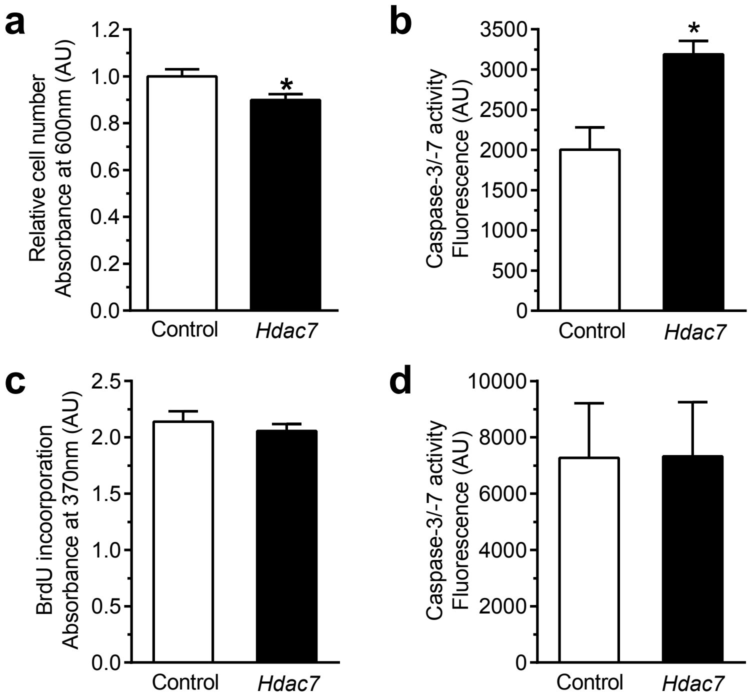

ESM Figure 1. *Hdac7* overexpression alters beta cell numbers by enhancing apoptosis. (**a**) *Hdac7* overexpression in clonal beta cells resulted in reduced cell numbers (n=6) and this reduction was due to increased apoptosis (**b**, n=6) rather than an impact on cellular proliferation (**c,** n=6). (**d**) The effect of *Hdac7* overexpression on apoptosis was also measured in rat islets. Two initial experiments showed that *Hdac7* overexpression had no effect on apoptosis in this setting and the experiments were abandoned. Data are presented as mean ± SEM. *p<0.05.

ESM Fig. 2

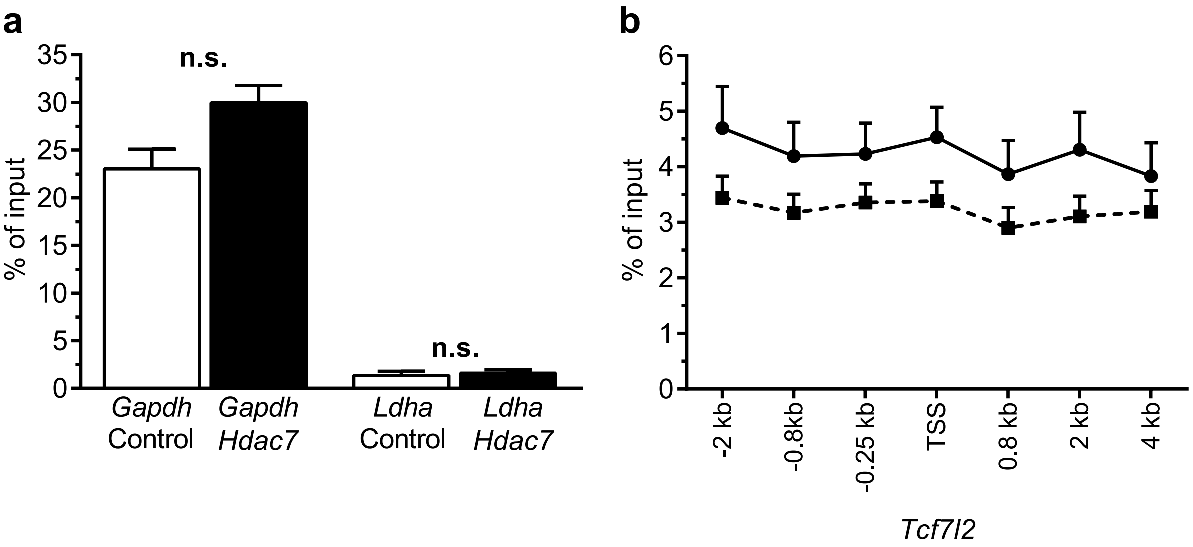

ESM Fig. 2 ChIP-qPCR analysis of *Tcf7l2* (**a**) ChIP-qPCR enrichment of H3K27ac at the promoter of positive control gene *Gapdh* and the negative control gene *Ldha*. (**b**) ChIP-qPCR enrichment of H3K27ac at -2kb to +4kb from the transcription start site of *Tcf7l2* gene (approximately -2kb, -0.8kb -0.25kb, TSS, 0.8kb, 2kb and 4kb). The data was analysed with paired T-tests (two tailed). Circles and solid line: control. Squares and dashed line: *Hdac7*. n=8.
